# c-KIT joins the TSC ToolKIT: a new driver of renal cystogenesis

**DOI:** 10.1038/s44321-025-00359-4

**Published:** 2025-12-22

**Authors:** Mark R Woodford, Dimitra Bourboulia, Mehdi Mollapour

**Affiliations:** 1 Department of Urology, SUNY Upstate Medical University, Syracuse, NY 13210 USA; 2 Upstate Cancer Center, SUNY Upstate Medical University, Syracuse, NY 13210 USA; 3 Department of Biochemistry and Molecular Biology, SUNY Upstate Medical University, Syracuse, NY 13210 USA

**Keywords:** Urogenital System

## Abstract

M. Mollapour and colleagues discuss the role of the proto-oncogene receptor tyrosine kinase c-KIT in Tuberous Sclerosis Complex (TSC) renal cystogenesis, as reported by M. Soleimani and colleagues, in this issue of EMBO Mol Med.

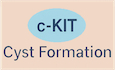

## Teaser

Tuberous Sclerosis Complex (TSC) is a multisystem disorder marked by benign tumors in brain, lung, and kidney. While the loss-of-function mutations in *TSC1* or *TSC2* have been known for decades, the molecular basis that converts these mutations into cystic kidney lesions has remained elusive. In this issue of *EMBO Molecular Medicine*, Zahedi and colleagues now uncover an unexpected culprit: the proto-oncogene receptor tyrosine kinase c-KIT. Their work identifies c-KIT as a pivotal driver of renal cystogenesis in TSC and suggests that its pharmacologic inhibition could complement existing mTOR-targeted therapy.

**Figure 1:**
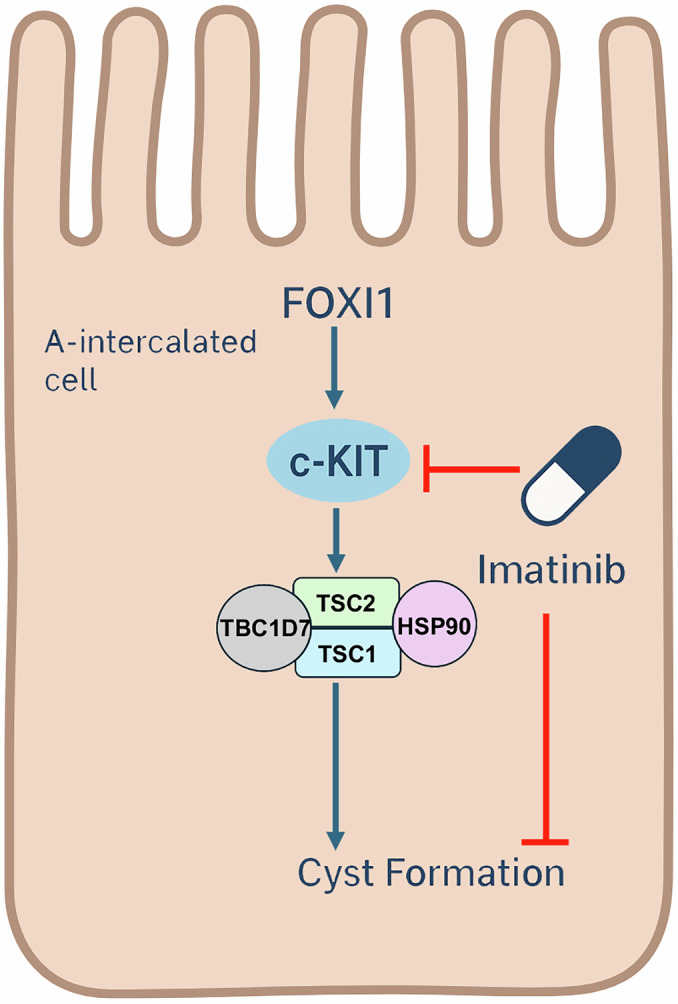
The FOXI1–c-KIT–TSC complex–Hsp90 axis regulates renal cystogenesis in Tuberous Sclerosis Complex (TSC). Schematic representation of the signaling cascade within A-intercalated (A-IC) cells of the kidney. The transcription factor FOXI1 induces the expression of the receptor tyrosine kinase c-KIT, which, upon activation, triggers downstream signaling that phosphorylates and inactivates TSC2 within the TSC1–TSC2–TBC1D7 complex, stabilized by the molecular chaperone Hsp90. This inactivation leads to hyperactivation of mTORC1, promoting A-IC proliferation and cyst formation. Pharmacologic inhibition of c-KIT by Imatinib (red blunt line) prevents this activation cascade and suppresses cystogenesis. The background silhouette outlines an A-intercalated cell to highlight the site of cyst initiation in TSC kidneys. The figure was prepared using BioRender software (https://biorender.com/).

Kidney cysts in TSC mouse models originate not from the expected principal cells but from A-intercalated cells (A-ICs), acid-secreting epithelia governed by the transcription factor FOXI1 (Barone et al, 2021). Eliminating *FOXI1* abolishes cyst formation, implicating an A-IC–specific program in disease. Zahedi et al, now connect this program to c-KIT activation (Zahedi, 2025). Transcriptomic profiling of *Tsc1*-deficient kidneys uncovered a strong up-regulation of *c-Kit* mRNA alongside *Foxi1* and other A-IC markers, which was ablated in cyst-free *Tsc1/Foxi1* double knockouts. Mechanistically, *FOXI1* overexpression in collecting-duct M-1 cells elevated c-KIT expression and boosted proliferation, mediated by phosphorylation of ERK1/2 and its downstream effector RSK1 (Zahedi, 2025). In *Tsc1*-knockout mice, activated p-Tyr–c-KIT localized to cyst epithelia that simultaneously displayed phospho-TSC2 (Ser 664/939/1798) and phosphorylation of ribosomal S6 protein (phospho-S6), hallmarks of mTORC1 activation. Genetic deletion of *c-Kit* completely prevented cystogenesis, normalizing ERK/AKT/RSK signaling and TSC2 phosphorylation. These findings establish a signaling hierarchy in which FOXI1 induces c-KIT, which then phosphorylates and inactivates TSC2, releasing mTORC1 from its inhibitory complex (Huang and Manning, 2008; Ma et al, 2005; Roux et al, 2004). Most TSC lesions, including angiomyolipomas and cortical tubers, follow the classic “two-hit” paradigm: loss of the wild-type *TSC* allele deactivates the TSC1–TSC2–TBC1D7 complex, unleashing mTORC1 (Henske et al, 1997). Renal cysts, however, retain both alleles and express intact TSC1/2 proteins. The Zahedi study clarifies how mTORC1 hyperactivation occurs without genetic loss through phosphorylation-mediated functional inactivation of TSC2 downstream of c-KIT (Fig. 1) (Zahedi, 2025). These contrasting mechanisms are described in cancer-prone TSC lesions, where loss of TSC1 destabilizes TSC2 via the Heat shock protein-70/Hsp90 chaperone network (Woodford et al, 2017). Together, these findings reveal that TSC pathobiology can arise from either chaperone-dependent protein instability or kinase-driven post-translational silencing of the complex. This “third pathway” mechanism may help explain other TSC manifestations that defy the loss-of-heterozygosity model. Encouragingly, oral administration of the clinically approved c-KIT inhibitor Imatinib Mesylate (Gleevec®) dramatically reduced cyst burden in *Tsc1*-deficient mice and curtailed p-S6 accumulation, without apparent toxicity (Fig. 1). This result positions Imatinib and possibly next-generation selective c-KIT blockers as candidates for repurposing in TSC-associated kidney disease, either alone or combined with rapalogs such as everolimus. Although, rapalogs remain the principal therapy for TSC but require lifelong administration and often show cyst regrowth upon discontinuation (Bissler et al, 2019). Dual targeting of mTORC1 and c-KIT could deliver more durable responses by attacking both the upstream driver and its effector. Yet Imatinib has a broad inhibition spectrum, including PDGFR, c-Abl, c-Src, and DDR1, which raises concerns about off-target effects in long-term pediatric use (Kim et al, 2014; Tsutsui et al, 2016). Future studies should evaluate more selective c-KIT inhibitors, dissect contributions of parallel RTKs, and determine whether KIT ligand (stem-cell factor) expression also rises in TSC kidneys. The distinction between TSC and autosomal-dominant polycystic kidney disease (ADPKD) is informative. ADPKD cysts arise from principal cells and depend on PKD1/2 mutations, while TSC cysts stem from A-ICs and FOXI1–c-KIT signaling (Fig. 1). Recognizing these divergent cellular origins underscores that “not all kidney cysts are created equal.” By linking a *proto*-oncogene classically associated with malignancy to a benign hereditary disorder, Zahedi et al, bridge cancer biology and cystic disease and offer an actionable therapeutic target already within the range of approved treatments.
